# Analyzing Sequential Betting with a Kelly-Inspired Convective-Diffusion Equation

**DOI:** 10.3390/e26070600

**Published:** 2024-07-15

**Authors:** Darrell Velegol, Kyle J. M. Bishop

**Affiliations:** 1 Department of Chemical Engineering, Penn State University, University Park, PA 16802, USA; 2 The Knowlecular Processes Company, State College, PA 16803, USA; 3 Department of Chemical Engineering, Columbia University, New York, NY 10023, USA; kyle.bishop@columbia.edu

**Keywords:** innovation portfolio, investment, bet, Kelly criterion, ruin

## Abstract

The purpose of this article is to analyze a sequence of independent bets by modeling it with a convective-diffusion equation (CDE). The approach follows the derivation of the Kelly Criterion (i.e., with a binomial distribution for the numbers of wins and losses in a sequence of bets) and reframes it as a CDE in the limit of many bets. The use of the CDE clarifies the role of steady growth (characterized by a velocity *U*) and random fluctuations (characterized by a diffusion coefficient *D*) to predict a probability distribution for the remaining bankroll as a function of time. Whereas the Kelly Criterion selects the investment fraction that maximizes the median bankroll (0.50 quantile), we show that the CDE formulation can readily find an optimum betting fraction *f* for any quantile. We also consider the effects of “ruin” using an absorbing boundary condition, which describes the termination of the betting sequence when the bankroll becomes too small. We show that the probability of ruin can be expressed by a dimensionless Péclet number characterizing the relative rates of convection and diffusion. Finally, the fractional Kelly heuristic is analyzed to show how it impacts returns and ruin. The reframing of the Kelly approach with the CDE opens new possibilities to use known results from the chemico-physical literature to address sequential betting problems.

## 1. Problem: Reframe the Kelly Approach as a Convective-Diffusion Equation

The purpose of this article is to analyze a sequence of independent bets by modeling it with a convective-diffusion equation (CDE). The CDE approach approximates innovation investing for the case where the projects are mostly independent (i.e., low correlation between successive events). “Innovation investments” differ from other investments like stocks, bonds, or commodities since there is no market for options or short selling with innovation [1].

Various models have been used to model sequential investments at both the portfolio and individual investment levels. At the portfolio level, early strategies focused on gaining high returns while avoiding risk. Modern portfolio theory (MPT) [2] introduced the concept of diversifying assets, and employs historical variance and covariance of the assets to assess risk. The Capital Asset Pricing Model (CAPM) followed from MPT, using a simple model with a parameter beta (β) that captures the risk of an asset relative to historical market outcomes. In 1973, the Black–Scholes model [1] was developed to analyze dynamic investment. The authors emphasized the role of options and shifted the focus from “risk aversion” to “risk neutrality”. They derived the Black–Scholes equation (BSE), which they called the “heat-transfer equation of physics”, and solved it for the price of an option that is risk neutral. Since the BSE has both drift and random walk terms, the equation they derived is actually more like the “convective-diffusion equation” than the “heat equation” or the “diffusion equation”.

Another model used to analyze sequential bets is the Kelly Criterion [3,4,5]. In 1956, Kelly examined the gambling or betting problem—or here, we would say the investing problem—with the goal of *maximizing* the growth of a bankroll rather than seeking risk neutrality. He considered a sequence of independent Bernoulli trials that accounts explicitly for the wins and losses of a bet, which have a given probability (*p*) of winning. The output from the Kelly model is the fraction (*f*) of current resources that should be bet in order to maximize future resources. The Kelly model has been applied to innovation investing [6]. The risk of the bet is specified by the value of *p* — which usually must be estimated for innovation investments — rather than from using the historical variance of the asset value as the BSE does. A champion of the Kelly approach to betting is Edward O. Thorp, a mathematician who had significant winnings both in the casino [7] and in the stock market [8]. Interestingly, based on Warren Buffet’s focus on long-term growth maximization instead of the avoidance of short-term losses, Davis et al. surmise that he is a Kelly Criterion bettor [9].

The Kelly Criterion has a number of variations. An important one is the use of “fractional Kelly”, in which one reduces risk by betting perhaps a half or a fourth of what the Kelly Criterion recommends [9]. Another is a Kelly Criterion with learning [10], which accounts for a change in parameters (e.g., a change in probability of winning) as expenditures are made. For example, as work is conducted on a project, the probability of success might rise. A third variation is Kelly with draw down control, determining how much to pull out as profit after each bet [11]. Other variations account for constraints in the betting, such as having to keep the total number of bets below some number. The focus of the Kelly Criterion and its variations has been on finding the betting fraction *f* that maximizes the median growth (0.50 quantile) for a given scenario.

Sometimes, however, we might seek a best “poor case” (similar to maximin) in order to avoid ruin, and sometimes we might seek a best “good case” to see what is possible. In this article, we remove the limitation of optimizing for the best median (i.e., 50th percentile or 0.50 quantile) return. Instead, we optimize our proportional betting parameter *f* so that the desired quantile γ is optimized. For example, we might conservatively want to optimize a poor-case scenario, say the 10th percentile (i.e., γ=0.10).

To do this, we reframe the Kelly approach as a CDE and provide solutions to the equation. The use of the CDE clarifies the role of steady growth (characterized by a velocity *U*) and random fluctuations (characterized by a diffusion coefficient *D*) to predict a probability distribution of the bankroll at any time. We analyze three main sub-problems:

- Optimal allocation *f* for the gamma (γ) quantile case, using our CDE;
- Optimal allocation *f* for a given “ruin” tolerance, using an absorptive boundary condition;
- Analysis of the return–risk trade-off for “fractional Kelly” betting.

## 2. Analysis Using the Convective-Diffusion Equation (CDE)

To analyze the questions above, we start by looking at a single investment, and we will proceed in several steps: (1) We will show that a binary win–loss game with proportional betting can be represented as a biased random walk. (2) We will model this random walk using a convective-diffusion equation (CDE). (3) We will use known CDE solutions to draw conclusions about the present problem.

This approach based on the CDE was used in the famous Black–Scholes model for pricing options [12]. They called their partial differential equation “the heat-transfer equation of physics”, although it also describes geometric Brownian motion including both deterministic drift (convection) and random movements (diffusion), which is why we call this equation the CDE. The Black–Scholes formula specifies conditions that determine the fair price of a risk-neutral option. Here, we take a different direction and provide solutions for the investment fraction that maximizes different objective functions, which depend on the predicted distribution of returns.

First, let us show that a binary win–loss game with proportional betting can be represented as a biased random walk. Starting with an initial bankroll $B_{0}$ for innovation, we want to predict a future bankroll *B* if we know the probability *p* of winning the bet, the winning odds *b* (such that if you bet $1 and win, you win *b* dollars plus your original $1), and the losing odds *a* (such that if you bet $1 and lose, you lose a fraction *a* of your original $1). We wager a fraction *f* of our bankroll on each such bet, holding the remainder in cash or allocating it for other bets. After *W* wins and *L* losses in *t* betting events (where t=W+L), the final bankroll $B_{t}$ is related to the initial bankroll $B_{0}$ by the following equation

(1) $$\frac{B_{t}}{B_{0}}={\left(1+fb\right)}^{W}{\left(1-fa\right)}^{L}$$

Taking the logarithm of both sides gives

(2) $$\ln B_{t}-\ln B_{0}=W\ln\left(1+fb\right)+L\ln\left(1-fa\right)$$

**Figure 1:**
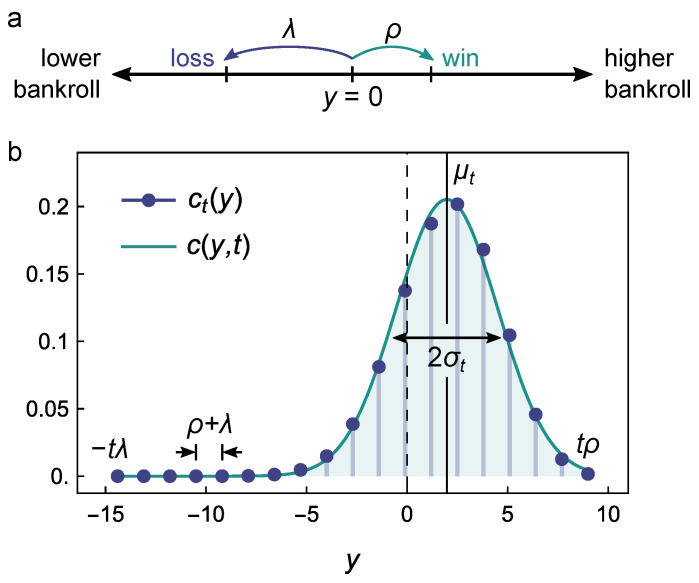
(**a**) Schematic showing the log bankroll $y=\ln(B/B_{0})$ increase by ρ to the right with a win (probability *p*) and decrease by λ to the left with a loss (probability q=1−p). In general, ρ≠λ. (**b**) Probability density $c_{t}\left(y\right)$ of Equation (6) after t=18 bets for parameters ρ=0.5, λ=0.8, and p=0.7. The discrete distribution (markers) spans from y=−tλ (all losses) to y=tρ (all wins) at regular intervals of ρ+λ. The distribution is annotated by the mean $\mu_{t}$ (vertical black line) and the standard deviation $\sigma_{t}$ (horizontal black line). The continuum distribution c(y,t) (teal curve) provides an accurate approximation when $\sigma_{t}\gg \rho +\lambda$ or, equivalently, pqt≫1.

We identify the distance $y_{t}$ between the log bankroll at time *t* and the log bankroll at t=0 as $y_{t}=\ln B_{t}-\ln B_{0}$. Wins and losses correspond to steps along the *y* axis to the right or left starting from $y_{0}=0$ (Figure 1a). When we win a bet, we move to the right by a step size ρ=ln(1+fb); when we lose a bet, we move to the left with a step size λ=−ln(1−fa)>0. Equation (2) now becomes

(3) $$y_{t}=W\rho -\left(t-W\right)\lambda$$

We denote the probability density of being at location *y* at (discrete) time *t* as $c_{t}\left(y\right)$. Initially, $c_{0}\left(y\right)=\delta \left(y\right)$, where δ(y) is the Dirac delta function since we start at y=0 by definition. The probability density is analogous to the concentration of a conserved quantity which is transported to the right or left by a random walk with drift. During a single betting event, the concentration at position *y* is updated due to steps to the right from position y−ρ (i.e., a win with probability *p*) and steps to the left from position y+λ (i.e., a loss with probability q=1−p). Mathematically, this updating is expressed as

(4) $$c_{t+1}\left(y\right)=pc_{t}\left(y-\rho \right)+qc_{t}\left(y+\lambda \right)$$

Starting from the initial concentration $c_{0}\left(y\right)=\delta \left(y\right)$, the repeated application of this updating rule leads to the following distributions after one and two betting events

(5) $$\begin{array}{c} \begin{array}{cc} c_{1}\left(y\right) & =p\delta (y-\rho )+q\delta (y+\lambda )\\ c_{2}\left(y\right) & =p^{2}\delta \left(y-2\rho \right)+2pq\delta \left(y-\rho +\lambda \right)+q^{2}\delta \left(y+2\lambda \right)\\ \; & \;\vdots \end{array} \end{array}$$

Following this pattern, the general solution can be expressed as

(6) ct(y)=∑W=0ttWpWqt−Wδy−Wρ+(t−W)λ

where the sum is evaluated over the possible number of wins *W* from 0 to *t*. The binomial coefficient describes the number of different ways of obtaining *W* wins in *t* bets. As illustrated in Figure 1b, this distribution is well summarized by its mean $\mu_{t}$ and variance $\sigma_{t}^{2}$

(7) $$\begin{array}{cc} \mu_{t} & =\int_{-\infty }^{\infty }yc_{t}\left(y\right)dy=\left(p\rho -q\lambda \right)t \end{array}$$

(8) $$\begin{array}{cc} \sigma_{t}^{2} & =\int_{-\infty }^{\infty }{\left(y-\mu_{t}\right)}^{2}c_{t}\left(y\right)dy=pq{\left(\lambda +\rho \right)}^{2}t \end{array}$$

Integrating the delta functions from Equation (6) in Equations (7) and (8) gives rise to summations, and the analytical summations give the final results of Equations (7) and (8). According to the central limit theorem, higher moments are increasingly negligible after many betting events *t*. For example, the third standardized moment known as skewness $\kappa_{t}$ decays with time *t* as

(9) $$\kappa_{t}=\int_{-\infty }^{\infty }{\left(\frac{y-\mu }{\sigma }\right)}^{3}c_{t}\left(y\right)dy=\frac{p-q}{\sqrt{pqt}}$$

For large *t*, the exact solution of Equation (6) is therefore well approximated by a normal distribution with mean $\mu_{t}$, standard deviation $\sigma_{t}$, and zero skewness. To accurately describe the discrete distribution of Equation (6) by a continuous normal distribution, we further require that the width of the distribution $\sigma_{t}$ be much larger than the step size ρ+λ. Substituting Equation (8) for the standard deviation, this condition is valid when pqt≫1.

Because the result from Equations (7) and (8) give a normal distribution for large *t*, the continuum approximation c(y,t) to the exact solution of Equation (6) satisfies the 1−D convective-diffusion equation (CDE) with velocity (sometimes called “drift”) *U* and diffusion coefficient *D* (to be determined)

(10) $$\frac{\partial c}{\partial t}=D\frac{\partial^{2}c}{\partial y^{2}}-U\frac{\partial c}{\partial y}$$

For the initial distribution c(y,t=0)=δ(y) on an unbounded domain (−∞<y<∞), the solution to Equation (10) is well known from diffusion theory [13]

(11) $$c\left(y,t\right)=\frac{1}{\sqrt{4\pi Dt}}\exp\left(-\frac{{\left(y-Ut\right)}^{2}}{4Dt}\right)$$

The result is a normal distribution with mean Ut and standard deviation $\sqrt{2Dt}$. By comparison to Equations (7) and (8) for the moments of the discrete solution, we identify the velocity and the diffusion coefficient to be

(12) $$U=p\rho -q\lambda ,\;D=\frac{1}{2}pq{\left(\lambda +\rho \right)}^{2}$$

Equations (11) and (12) give the probability density c(y,t) for being at any value of *y* (i.e., the log bankroll) at some dimensionless time *t*. This result is for a sequence of binary win–lose outcomes. We have added a result in Appendix A for the case when there are more than two possible outcomes to consider. It is important to remember that the given solution depends implicitly on our choice of *f* since the velocity *U* and diffusivity *D* both depend on *f* by way of the step sizes ρ=ln(1+fb) and λ=−ln(1−fa). In the next section, we will explore how we should choose an optimal betting fraction *f* for different candidate objective functions.

## 3. Optimizing f

Our choice of betting fraction *f* influences the distribution of outcomes c(y,t) for a specified number of bets *t*, each with win probability *p*, loss ratio *a*, and payback ratio *b*. Choosing an optimal value of *f* depends on our risk preferences, which are described implicitly by our choice of objective function to be maximized. Here, we consider three different objectives: (1) optimizing the mean bank roll, (2) optimizing the median bankroll, and (3) optimizing the γth quartile of the bankroll. In the next section, we give an alternative perspective, which is to avoid ruin.

### 3.1. Mean Bankroll

Using the continuum approximation, the expected bankroll $B=B_{0}e^{y}$ is given by

(13) $$E\left(B\right)=\int_{-\infty }^{\infty }B_{0}e^{y}c\left(y,t\right)dy=B_{0}e^{(U+D)t}$$

Provided that U+D>0, the mean bankroll is expected to grow exponentially with time due both to convective transport with velocity *U* and diffusive transport with diffusivity *D*. We can optimize our expected rate of return by choosing the betting fraction *f* that maximizes U+D. Substituting Equation (12) for *U* and *D*, the objective function to be maximized can be written as

(14) $$U+D=q\ln\left(1-fa\right)+p\ln\left(1+fb\right)+\frac{1}{2}pq{\left[\ln(1+fb)-\ln(1-fa)\right]}^{2}$$

For any values of *a*, *b*, *p*, and q=1−p, this function is strictly monotonic in *f*, increasing from zero at f=0 and approaching infinity as f→1/a. Thus, to maximize the mean bankroll, we should let f=1 (i.e., “bet it all”), or if possible, leverage our position for f>1. If we allocate f→1/a such that ln(1−fa)→−∞ (i.e., we “risk losing it all”), the velocity *U* approaches negative infinity while the diffusivity *D* approaches positive infinity even faster. This rapid diffusion of the distribution driven by the large step size of the random walk leads to extreme outcomes, some of which are so lucrative as to shift the *mean* outcome to be favorable for this aggressive strategy. However, as with the St. Petersburg paradox, few would “bet it all” in this way.

### 3.2. Median Bankroll

The median bankroll *B* corresponds to the median of the log bankroll, which is simply y=Ut. The median does not involve contributions from diffusion, which acts symmetrically to broaden the distribution c(y,t). We can optimize the median rate of return by choosing the betting fraction *f* that maximizes *U*. The optimum betting fraction *f* satisfies the conditions

(15) $$\frac{dU}{df}=0\;\mathrm{implies}\;f_{\mathrm{KC}}=\frac{p}{a}-\frac{q}{b}$$

This result corresponds to the original Kelly Criterion $f_{\mathrm{KC}}$ described previously [3,6].

### 3.3. Specified Quantile

We now optimize the growth of $y=\ln(B/B_{0})$ for any quantile result. We will designate the γth quantile result as $y_{\gamma }$. So γ=0.25 is the 0.25 quantile (i.e., 25th percentile or first quartile); γ=0.50 is the 0.50 quantile (i.e., 50th percentile or median); and γ=0.75 is the 0.75 quantile (i.e., 75th percentile or third quartile). At any given time *t*, we can integrate the probability c(y,t) from y→−∞ (which has probability density c→0) up to some as yet unknown value $y_{\gamma }$, which defines the quantile:

(16) $$\int_{-\infty }^{y_{\gamma }}c\left(y,t\right)dy=\gamma$$

Using the properties of the normal distribution, this integral can be evaluated for the continuum solution of Equation (11) to obtain

(17) $$\frac{1}{2}\left[1+\mathrm{erf}\left(\frac{y_{\gamma }-Ut}{\sqrt{4Dt}}\right)\right]=\gamma$$

where erf(x) is the error function of *x*. Thus, for any quantile, we find the value of $y_{\gamma }$ by rearranging Equation (17) as

(18) $$y_{\gamma }\left(t\right)=Ut+\sqrt{4Dt}\;{\mathrm{erf}}^{-1}\left(2\gamma -1\right)$$

where ${\mathrm{erf}}^{-1}\left(x\right)$ is the inverse error function of *x*. The first term, describing convective transport, increases (U>0) or decreases (U<0) linearly with time depending on the sign of *U*. The second term, describing diffusive transport, increases (γ>0.5) or decreases (γ<0.5) with time as $t^{1/2}$ depending on the quantile γ. We are interested in identifying winning outcomes for which $y_{\gamma }\left(t\right)>0$ such that the γth quantile of our bankroll increases from its starting value $B_{0}$. Such outcomes are assured when U>0 and γ>0.5 but are also possible when U<0 or γ<0.5 for certain time windows. When U>0 and γ<0.5, convection acts to increase $y_{\gamma }$ while diffusion acts to decrease it. Winning outcomes are achieved for times *t* greater that the critical value

(19) $$t_{\gamma }=\frac{4D}{U^{2}}\;{\left[{\mathrm{erf}}^{-1}\left(2\gamma -1\right)\right]}^{2}$$

By contrast, when U<0 and γ>0.5, convection acts to decrease $y_{\gamma }$, while diffusion acts to increase it. Winning outcomes are achieved for times $t<t_{\gamma }$. These four scenarios are summarized in Table 1.

To find the optimal allocation *f* towards the bet at any quantile, we set

(20) $$\frac{dy_{\gamma }}{df}=0$$

and solve for *f*. In general, this equation must be solved numerically; however, we can gain insight from the limiting behaviors at short and long times. At long times ($t\gg t_{\gamma }$), convection is dominant, and we recover the Kelly result of Equation (15). At short times ($t\ll t_{\gamma }$) when diffusion is dominant, maximizing $y_{\gamma }$ with respect to γ is identical to maximizing the diffusivity *D*

(21) $$\frac{dD}{df}=0\;\mathrm{implies}\;f\to 1$$

By placing big bets (f→1), we increase the step size ρ+λ of our random walk and thereby accelerate the rate of diffusive transport. Note, however, that this conclusion assumes that the continuum approximation remains valid (i.e., when ${\left(pq\right)}^{-1}\ll t\ll t_{\gamma }$) and that repeated betting is possible without “going bust” (see below).

**Figure 2:**
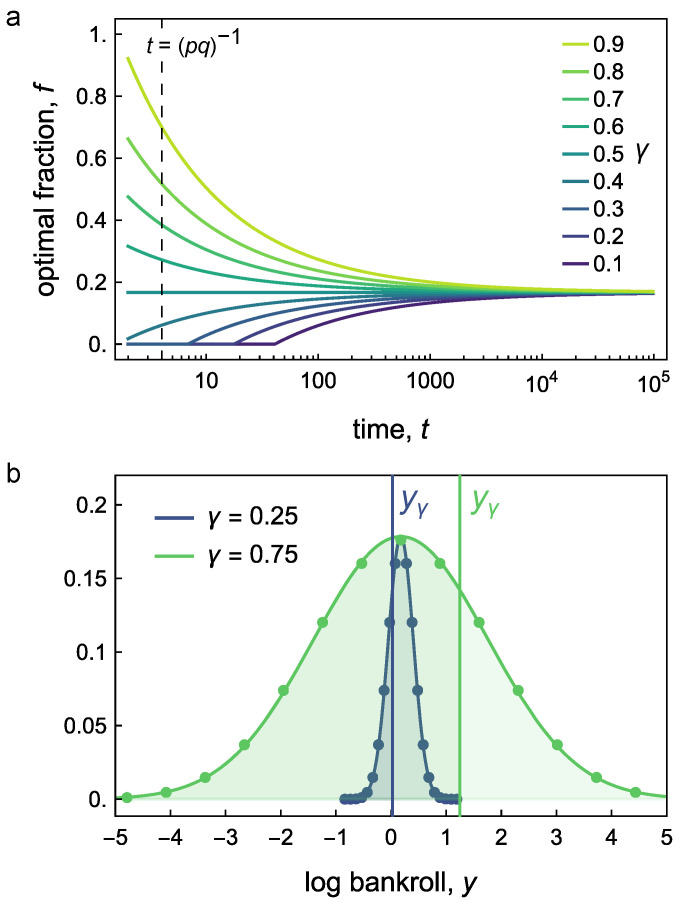
(**a**) Optimal betting fraction *f* as a function of time *t* for different quantiles γ obtained by the numerical solution of Equation (20). The parameters are p=0.5, a=1, and b=1.5 corresponding to the original game discussed in an earlier paper [6]. The continuum approximation is valid to the right of the vertical dashed line, $t\gg {\left(pq\right)}^{-1}$. (**b**) Distributions of the log bankroll *y* after t=20 bets for optimal betting fractions *f* corresponding to the first and third quartiles: γ=0.25 and γ=0.75. The parameters are those from part (**a**).

As an example, let us consider the optimal values of *f* for various quantiles γ and times *t* for the specific parameters p=0.5, a=1, and b=1.5 corresponding to the original game discussed in an earlier paper [6]. Figure 2 plots the optimal fraction *f* as a function of time *t* for different quantiles γ. At long times, convection dominates, and we recover the Kelly Criterion of Equation (15)—here, $f_{\mathrm{KC}}=1/6$. At shorter times, the optimal fraction is greater than $f_{\mathrm{KC}}$ for γ>0.5 and less than $f_{\mathrm{KC}}$ for γ<0.5. The former corresponds to the optimist’s strategy which chooses *f* to maximize good outcomes. By contrast, the pessimist’s strategy (γ<0.5) chooses *f* to maximize poor outcomes. Diffusion is therefore favorable to the optimist but unfavorable to the pessimist.

## 4. Avoiding Ruin

If our bankroll becomes too small during the course of repeated betting events, it may become impossible to make further gambles. We have “gone bust”. We define a ruin tolerance

(22) $$r=B_{r}/B_{0}<1$$

such that we can make no further bets when the bankroll falls below $B_{r}$. The possibility of ruin can be described by modifying the random walk to include an absorbing boundary at y=−ℓ, where ℓ=ln(1/r) is the distance from the initial condition to ruin. When the random walker steps below this ruin length, betting ceases and the walk ends. (We could also place an “absorbing boundary” at a position y>0, in which case we stop when we have won enough to be satisfied rather than having lost enough.) In our continuum CDE description of Equation (10), the possibility of ruin is described by the following boundary condition

(23) c(−ℓ,t)=0

**Figure 3:**
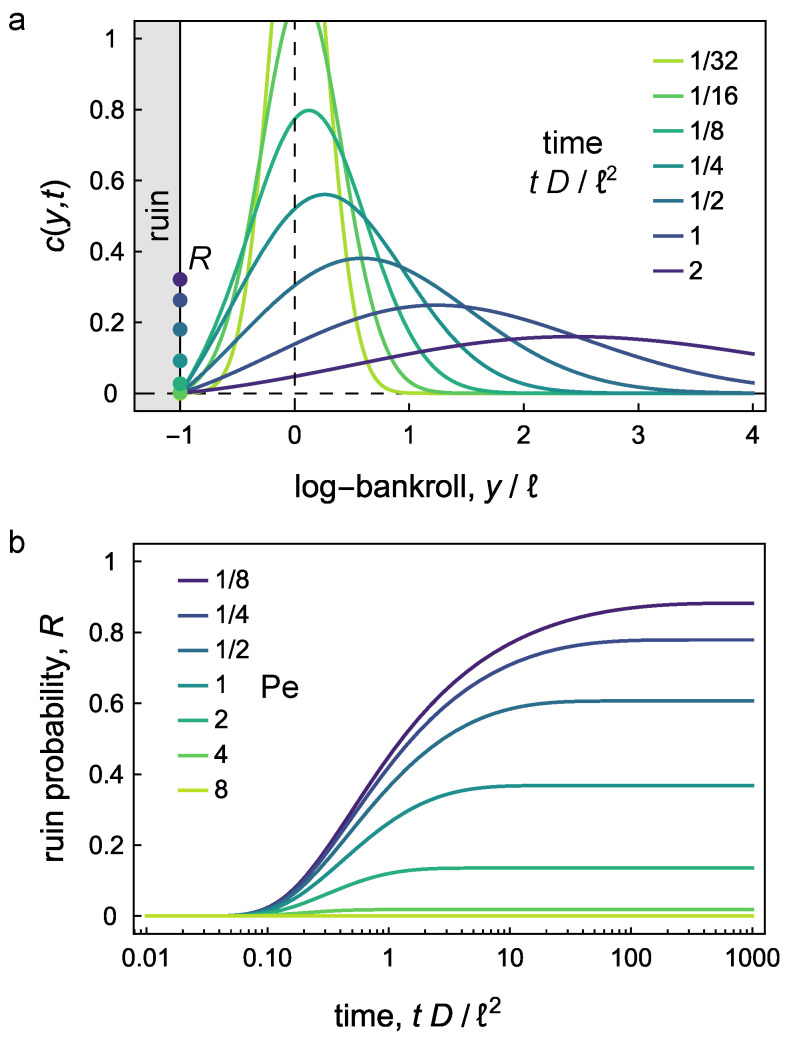
(**a**) Transient distribution c(y,t) for the log bankroll *y* for Péclet number, Pe=ℓU/D=1. The log bankroll is scaled by the ruin length *ℓ*; time is scaled by the diffusive time scale $\ell^{2}/D$. Markers at the absorbing boundary denote the transient ruin probability *R*. (**b**) Accumulated ruin probability *R* of Equation (27) as a function of time for different Péclet numbers Pe=ℓU/D>0. Time is scaled by the diffusive time scale $\ell^{2}/D$. The values asymptote for any given value of Pe, to the value given in Equation (28).

Starting from the initial distribution c(y,0)=δ(y), we can solve the convection–diffusion equation subject to Equation (23) to obtain

(24) $$c\left(y,t\right)=\frac{1}{\sqrt{4\pi Dt}}\exp\left(-\frac{{\left(y-Ut\right)}^{2}}{4Dt}\right)\left[1-\exp\left(\frac{\ell (y+\ell )}{Dt}\right)\right]$$

on the domain −ℓ≤y<∞ (Figure 3a; see Supplementary Materials for derivation).

Using this solution, we can evaluate the ruin rate *J*, which characterizes the flux of probability through the absorbing boundary at y=lnr

(25) $$J\left(t\right)=-{\left(Uc-D\frac{dc}{dy}\right)}_{y=-\ell }$$

Substituting Equation (24), the ruin rate can be expressed as

(26) $$J\left(t\right)=\frac{\ell }{\sqrt{4\pi Dt^{3}}}\exp\left(-\frac{{\left(\ell +Ut\right)}^{2}}{4Dt}\right)$$

The quantity J(t)dt describes the probability of ruin within the differential time interval *t* to t+dt. We can integrate the ruin rate with respect to time from 0 some later time *t* to obtain the accumulated probability of ruin as

(27) $$R\left(t\right)=\frac{1}{2}\left[\mathrm{erfc}\left(\frac{\ell +Ut}{\sqrt{4Dt}}\right)+e^{-\ell U/D}\;\mathrm{erfc}\left(\frac{\ell -Ut}{\sqrt{4Dt}}\right)\right]$$

At long times, the ruin probability approaches a constant value that depends on the dimensionless Péclet number Pe=ℓU/D, which characterizes the relative rates of convection and diffusion over the ruin length *ℓ*. When convection transports the distribution *away* from the absorbing ruin boundary (i.e., when U>0), the asymptotic ruin probability is

(28) $$R_{\infty }=R\left(t\to \infty \right)=e^{-\mathrm{Pe}}=r^{U/D}$$

For large Péclet numbers (Pe≫4), this asymptotic limit is achieved for times t≫ℓ/U, which is the time required to convect the distance *ℓ*. For small Péclet numbers (Pe≪4), the asymptotic behavior of Equation (28) is realized when $t\gg 4D/U^{2}$ beyond which convection is dominant compared to diffusion. By contrast, when convection transports the distribution *towards* the absorbing boundary (i.e., when U<0), ruin becomes inevitable at long times.

Figure 3 plots the ruin probability as a function of time for “winning” investments with U>0. Ruin can be avoided when the Péclet number is large Pe≫1—for example, by increasing the ratio U/D or by lowering our ruin tolerance *r* (thereby increasing *ℓ*). If, however, we seek to *minimize* the ruin probability by varying the betting fraction *f*, we are led to the conservative strategy in which f→0. That is, we can avoid ruin by risking nothing. Alternatively, we can use the ruin probability as a constraint on the optimization of other objective functions such as the γ-quantile described above.

**Figure 4:**
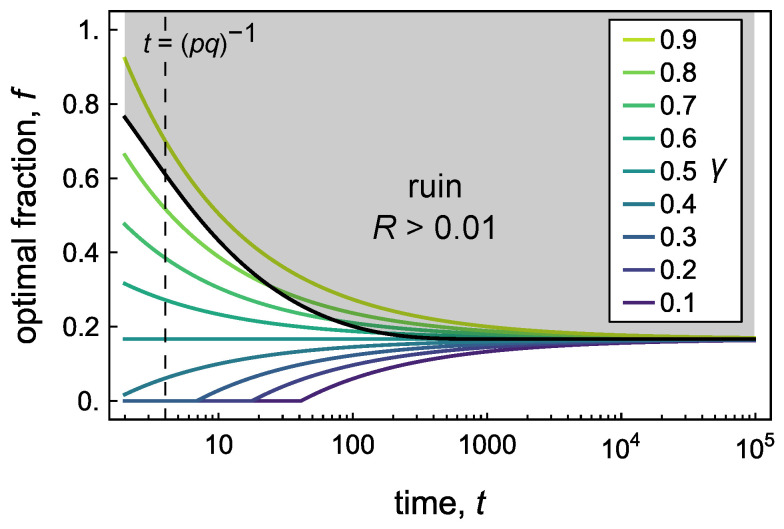
Optimal betting fraction *f* as a function of time *t* for different quantiles γ reproduced from Figure 2a for p=0.5, a=1, and b=1.5. The black curve shows the ruin boundary, above which the ruin probability is greater than a specified value $R_{\max}=0.01$. The ruin tolerance r=0.00969 is chosen to permit the Kelly solution $f_{\mathrm{KC}}=1/6$ at long times. For short times, one might prefer to use a “fractional Kelly Criterion” to increase safety.

Figure 4 shows how the ruin probability can be used to constrain the optimization of the γ-quantile. Here, we specify a maximum ruin probability of $R_{\max}$ and seek investment strategies for which $R\left(t\right)<R_{\max}$. When the chance of ruin is small ($R_{\max}\ll 1$), the absorbing boundary solution of Equation (24) is well approximated by the unbounded solution of Equation (11). The maximum ruin length $\ell_{\max}$ and the associated ruin tolerance $r_{\max}$ can then be determined from Equation (28) as

(29) $$\ell_{\max}=\ln\left(1/r_{\max}\right)=\frac{D}{U}\ln\left(1/R_{\max}\right)$$

where the velocity *U* and diffusivity *D* are evaluated at the Kelly betting fraction $f_{\mathrm{KC}}$. This assignment ensures that the optimal *f* for a specified quantile γ will avoid ruin at long times t→∞. For the present example with $R_{\max}=0.01$, this condition implies that $r_{\max}=0.00969$ and ℓ=4.64. At intermediate times *t*, the betting fraction *f* must be set below some time-dependent value to ensure the desired ruin probability $R\leq R_{\max}$ (Figure 4, black curve). This ruin constraint prohibits some of the riskier strategies for quantiles γ>0.5.

When the ruin probability is not negligibly small, it is no longer possible to decouple the optimization of the betting fraction *f* from the enforcing of the ruin constraint. The probability density c(y,t) of Equation (11) which neglects ruin becomes qualitatively different from that of Equation (24), which accounts for it. In this case, we must search for the betting fraction *f* that optimizes the γ-quantile of Equation (24) subject to the ruin constraint that $R\left(t\right)<R_{\max}$. This problem can be solved numerically but is not considered further here.

## 5. Fractional Kelly

Betting a fraction of the Kelly Criterion $f_{\mathrm{KC}}$—or “Fractional Kelly”—is a common heuristic for reducing risk and avoiding ruin while maintaining a high return [9]. Although fractional Kelly is sometimes viewed as an ad hoc correction, we can use our analysis to relate this betting policy to that associated with (1) a particular quantile or (2) a particular ruin rate or probability. Figure 2a can be used to relate a fractional Kelly policy to the corresponding quantile policy for a given time horizon *t* (for better precision, solve Equation (20) for γ specifying *f* equal to fractional KC). For example, in our game from this paper, $f_{\mathrm{KC}}=1/6$, which corresponds to γ=0.5. If we choose “half Kelly”, that is f=1/12, this gives added safety at small times, corresponding to γ=0.412 at t=5 or γ=0.264 at t=40.

Alternatively, and perhaps of more value, we might want to relate some fraction of the KC to the median growth rate and ruin probability. We can use the parameters of a game (in this article, p=q=0.5, a=1, b=1.5) to find ρ and λ, and thus *U* and *D*. We use Equation (1) to evaluate the median growth per flip, and subtract 1 = 100% to get the growth rate. We can then use the *U* and *D* in Equation (28) to obtain the asymptotic ruin probability $R_{\infty }$ or Equation (27) to obtain R(t) at any earlier time. Here we’ll use a ruin tolerance of r=0.01.

For the full Kelly Criterion, $f_{\mathrm{KC}}=1/6$, giving U=0.0204 and D=0.0206. The resulting Péclet number is Pe=4.57. This gives the median return of 1.0206/flip, or a growth rate of 2.06% per flip. The asymptotic ruin probability $R_{\infty }$ is $1.03\times 10^{-2}$. For “half Kelly”, f=1/12, giving U=0.0154 and D=0.00524, which gives Pe=13.52. The median return per flip is 1.0155, or a growth of 1.55% per flip (75% as much as full Kelly). $R_{\infty }$ drops considerably, to $1.35\times 10^{-6}$. For “quarter Kelly”, f=1/24, giving Pe=31.3. The median return per flip is 1.0091, or a growth of 0.91% per flip (44% as much as full Kelly), while $R_{\infty }$ drops to $2.67\times 10^{-14}$. For this game, the quarter Kelly policy would almost never reach ruin, and yet give almost half the growth rate.

## 6. Conclusions

The purpose of this article is to analyze a sequence of independent bets by modeling it with the convective-diffusion equation (CDE). This provides a model for innovation investing when the projects are independent. We have combined the CDE formulation with known results from the physico-chemical literature, to produce solutions for the growth of a bankroll due to sequential innovation bets, as well as the entropic fluctuations. By applying an absorptive boundary condition, we have derived a readily usable result for the ruin rate in terms of the system Peclet number. Finally, we have shown how the previously ad hoc fractional Kelly heuristic affects returns and risk.

The fluctuations resulting from the diffusive effects of innovation investments increase the variance of the final outcomes. Thus, while previous Kelly Criterion modeling emphasized the best median result—which is independent of diffusion—our work shows how diffusion can lead to very favorable outcomes as well as unfavorable outcomes for the same underlying scenario. The quantitative intuition gained from our results can guide professionals away from situations where the possibility of ruin is greater than desired.

This work can, in the future, be extended in several directions by pursuing the following questions: (1) Can we use the analytical machinery we have derived to create a practical tool for guiding innovation investments and portfolio management? (2) Can the present technical analysis impact actual companies, which have internal psychological and political forces? And perhaps most immediately, (3) what other CDE solutions might be used to provide new insights into innovation investing? By framing our problem in terms of the CDE, we aim to inspire others to extend insights from the chemico-physical literature to gain an edge in sequential betting, perhaps including innovation investing, as well as other aspects of life.

## Figures and Tables

**Table 1:** The possibility of winning outcomes $y_{\gamma }>0$ depends on the velocity *U*, the quantile γ, and the number of betting events *t*. We denote losing outcomes with the label “gambler” (U<0), and winning outcomes (U>0) with the label “investor”. We use the labels “pessimist” and “optimist” to denote objective functions that emphasize worse outcomes (γ<0.5) and better outcomes (γ>0.5), respectively.

	Gambler (U<0)	Investor (U>0)
Pessimist (γ<0.5)	wins for **no** *t*	wins for **long** $t>t_{\gamma }$
Optimist (γ>0.5)	wins for **short** $t<t_{\gamma }$	wins for **all** *t*

## Data Availability

Data (calculated) are contained within the article.

## Supplementary Materials

The following supporting information can be downloaded at: https://www.mdpi.com/article/10.3390/e26070600/s1. See Supporting Information for (1) the derivation of Equation (24) for convection–diffusion on a semi-infinite domain, and (2) the distribution for the log bankroll for a sequence of betting events with more than two outcomes.

## Appendix A. Generalization to More than Two Outcomes

In this article, we have considered a sequence of binary win–lose outcomes, each with an associated probability (*p* and *q*) and payback odds (*a* and *b*). However, when we make an investment, we often have more than two possible outcomes to consider. Here, we consider a number Ω of possible outcomes for each *t* independent betting events. Each outcome *i* is associated with a payback odds $v_{i}$ and occurs with probability $\phi_{i}\geq 0$ such that $\sum_{i=1}^{\Omega }\phi_{i}=1$. Note that favorable outcomes (“wins”) have positive odds $v_{i}>0$, while unfavorable outcomes (“losses”) have negative odds $v_{i}<0$. For example, $v_{1}=-a$ and $v_{2}=b$ for the two outcome scenarios described in earlier sections.

For a single event, the expectation of the log bankroll $y=\ln B/B_{0}$ is given by

(A1) $$\mu_{1}=\sum_{i=1}^{\Omega }\phi_{i}\ln\left(1+fv_{i}\right)$$

Similarly, the variance of the log bankroll after one game is given by

(A2) $$\begin{array}{c} \begin{array}{cc} \sigma_{1}^{2} & =\sum_{i=1}^{\Omega }\phi_{i}{\left(\ln\left(1+fv_{i}\right)-\mu_{1}\right)}^{2}=\sum_{i=1}^{\Omega }\phi_{i}\ln{\left(1+fv_{i}\right)}^{2}-{\left(\sum_{i=1}^{\Omega }\phi_{i}\ln\left(1+fv_{i}\right)\right)}^{2} \end{array} \end{array}$$

These expressions simplify to Equations (7) and (8) for the case of Ω=2 when $v_{1}=-a$, $v_{2}=b$, $\phi_{1}=q$, and $\phi_{2}=p$. The log bankroll after *t* such betting events corresponds to the sum of *t* independent random variables each with mean $\mu_{1}$ and variance $\sigma_{1}^{2}$. The mean and variance of this sum are simply $\mu_{t}=t\mu_{1}$ and $\sigma_{t}^{2}=t\sigma_{1}^{2}$. After many betting events, the central limit theorem (CTL) implies that the log bankroll *y* is well approximated by a normal distribution with mean $\mu_{t}$ and standard deviation $\sigma_{t}$. By comparison to the CDE solution of Equation (11), the velocity and the diffusivity are identified as $U=\mu_{1}$ and $D=\sigma_{1}^{2}/2$. The exact distribution for the log bankroll valid for all *t* is described in the Supporting Information.
