# Supplementary material for: Analyzing Sequential Betting with a Kelly-Inspired Convective-Diffusion Equation
Source: Entropy (Basel). 2024 Jul 15;26(7):600. doi: 10.3390/e26070600 (PMC11276101; doi:10.3390/e26070600)
Supplement: Supplementary file 1 [file entropy-26-00600-s001.zip › entropy-2995584-supplementary-english.pdf]

Supporting Information: Analyzing sequential betting with a Kelly-style convective-diffusion equation

Darrell Velegol[*,†,‡] and Kyle J. M. Bishop[*,¶]

†*Department of Chemical Engineering, Penn State University, University Park PA 16802 USA*

‡*The Knowlecular Process Company, State College PA 16803 USA*

¶*Department of Chemical Engineering, Columbia University, New York NY 10023 USA*

E-mail: velegol@psu.edu; kyle.bishop@columbia.edu

Convection–diffusion on a semi-infinite domain

Here, we consider the following convection–diffusion problem

$$\frac{\partial c}{\partial t} = D\frac{\partial^2 c}{\partial y^2} - U\frac{\partial c}{\partial y} \tag{S1}$$

subject to initial and boundary conditions

$$c(y,0) = \delta(y) \tag{S2}$$

$$c(\ln r, t) = 0 \tag{S3}$$

$$c(\infty, t) = 0 \tag{S4}$$

The probability density $c(y,t)$ is initially localized at $y = 0$ and evolves in time due to convection with velocity U and diffusion with diffusivity D. An absorbing boundary condition at $y = \ln r$ describes the effect of "ruin" with a ruin tolerance of $r = B/B_0$.

Scaling length by $-\ln r$ and time by $(\ln r)^2/D$, this problem can be recast in dimensionless form as

$$\frac{\partial c}{\partial t} = \frac{\partial^2 c}{\partial y^2} - \mathrm{Pe}\frac{\partial c}{\partial y}, \quad c(y,0) = \delta(y), \quad c(-1,t) = 0, \quad c(\infty,t) = 0 \tag{S5}$$

where $\mathrm{Pe} = -\ln r\, U/D$ is the (dimensionless) Péclet number characterizing the relative rates of convection and diffusion. This problem admits the following solution

$$c(y,t) = \frac{1}{\sqrt{4\pi t}} \exp\left(-\frac{(y - \mathrm{Pe}\, t)^2}{4t}\right)\left[1 - \exp\left(-\frac{1+y}{t}\right)\right] \tag{S6}$$

on the domain $-1 < y < \infty$ and $t > 0$.

Derivation. The solution (S6) can be obtained using Laplace transforms as follows. First, we introduce the Laplace transform

$$\hat{c}(y,s) = \int_0^\infty c(y,t)dt \tag{S7}$$

Applying this transform to the governing equation (S5) and making use of the initial condition, we obtain the following ordinary differential equation

$$s\hat{c} - \delta(y) = \frac{\partial^2 \hat{c}}{\partial y^2} - \mathrm{Pe}\frac{\partial \hat{c}}{\partial y} \tag{S8}$$

This equation admits the general solution

$$\begin{aligned}\hat{c}(y,s) = {} & C_+ e^{\left(\mathrm{Pe}+\sqrt{\mathrm{Pe}^2+4s}\right)y} + C_- e^{\left(\mathrm{Pe}-\sqrt{\mathrm{Pe}^2+4s}\right)y} \\ & - \frac{2e^{\frac{1}{2}\mathrm{Pe}\,y}\sinh\left(\frac{1}{2}\sqrt{\mathrm{Pe}^2+4s}\,y\right)}{\sqrt{\mathrm{Pe}^2+4s}}\Theta(y)\end{aligned} \tag{S9}$$

where $\Theta(y)$ is the Heaviside step function, and $C_\pm$ are integration constants. Applying the boundary conditions at $y=-1$ and $y \to \infty$, the solution becomes

$$\begin{aligned}\hat{c}(y,s) = \frac{e^{\frac{1}{2}\left(\mathrm{Pe}-\sqrt{\mathrm{Pe}^2+4s}\right)y}}{\sqrt{\mathrm{Pe}^2+4s}} \Bigg[& \left(1 - e^{-\sqrt{\mathrm{Pe}^2+4s}}\right)\Theta(y) + \\ & + \left(e^{\sqrt{\mathrm{Pe}^2+4s}\,y} - e^{-\sqrt{\mathrm{Pe}^2+4s}}\right)\left(1-\Theta(y)\right)\Bigg]\end{aligned} \tag{S10}$$

Taking the inverse Laplace transform of this expression yields the final solution (S6).

Ruin rate

The ruin rate J corresponds to the probability flux into the absorbing boundary at $y = -1$

$$\begin{aligned} J &= -\left(\mathrm{Pe}\, c - \frac{\partial c}{\partial y}\right)_{y=-1} \\ &= \frac{1}{\sqrt{4\pi t^3}} \exp\left(-\frac{(1+\mathrm{Pe}\, t)^2}{4t}\right) \end{aligned} \tag{S11}$$

Integrating this ruin rate with respect to time gives the probability of "going bust" within a finite time t

$$R(t) = \frac{1}{2}\left[\mathrm{erfc}\left(\frac{1+\mathrm{Pe}\, t}{\sqrt{4t}}\right) + e^{-\mathrm{Pe}}\,\mathrm{erfc}\left(\frac{1-\mathrm{Pe}\, t}{\sqrt{4t}}\right)\right] \tag{S12}$$

The asymptotic probability of "going bust" at long times is

$$R(t \to \infty) = e^{-\mathrm{Pe}} \tag{S13}$$

Generalization to more than two outcomes

As in Appendix A, we consider a sequence of t betting events each with Ω possible outcomes. We denote the number of "wins" of type i as W_i with the condition $\sum_{i=1}^{\Omega} W_i = t$; each outcome i is associated with a payback odds v_i. The resulting bankroll B can then be expressed as

$$\frac{B}{B_0} = \prod_{i=1}^{\Omega} (1 + f v_i)^{W_i} \tag{S14}$$

This equation for multiple outcomes represents a generalization of Eq 1 for two outcomes. The parameters $-a$ and b from the earlier sections are replaced with v_i for the ith outcome; note that positive outcomes have $v_i > 0$, while negative outcomes have $v_i < 0$. Generalizing Eq 2, the log bankroll y for multiple outcomes becomes

$$y = \ln\left(\frac{B}{B_0}\right) = \sum_{i=1}^{\Omega} W_i \ln(1 + f v_i) \tag{S15}$$

We now consider that each outcome i occurs with some probability $\phi_i \geq 0$ such that $\sum_{i=1}^{\Omega} \phi_i = 1$. The probability of obtaining the set of outcomes $\{W_1, W_2, \ldots W_\Omega\}$ in t identical betting events is given by the multinomial distribution

$$f(W_1, W_2, \ldots W_\Omega) = \frac{t!}{W_1! W_2! \ldots W_\Omega!} \prod_{i=1}^{\Omega} \phi_i^{W_i} \tag{S16}$$

The corresponding distribution for the log bankroll is

$$c_t(y) = \sum_{W_1, W_2, \ldots, W_\Omega} f(W_1, W_2, \ldots, W_\Omega) \delta \left(y - \sum_{i=1}^{\Omega} W_i \ln(1 + f v_i) \right) \tag{S17}$$

where the first sum is over the possible outcomes $W_i \geq 0$ satisfying $\sum_{i=1}^{\Omega} W_i = t$. We can

evaluate the mean μ_t of the log bankroll y as

$$\begin{aligned}
\mu_t &= \int_{-\infty}^{\infty} c_t(y) y \, dy \\
&= \sum_{\{W_k\}} f(\{W_k\}) \sum_{i=1}^{\Omega} W_i \ln(1 + f v_i) \\
&= \sum_{i=1}^{\Omega} \ln(1 + f v_i) \sum_{\{W_k\}} f(\{W_k\}) \, W_i \\
&= t \sum_{i=1}^{\Omega} \phi_i \ln(1 + f v_i)
\end{aligned} \tag{S18}$$

where $\{W_k\}$ denotes the set of outcomes $W_k \geq 0$ such that $\sum_{k=1}^{\Omega} W_k = t$, and we have made use of the known mean of the multinomial distribution $E(W_i) = t\phi_i$. Similarly, the variance σ_t^2 of the log bankroll is

$$\begin{aligned}
\sigma_t^2 &= \int_{-\infty}^{\infty} c_t(y)(y - \mu_t)^2 dy \\
&= \sum_{\{W_k\}} f(\{W_k\}) \left(\sum_{i=1}^{\Omega} W_j \ln(1 + f v_j) - t \sum_{i=1}^{\Omega} \phi_i \ln(1 + f v_i) \right)^2 \\
&= \sum_{i=1}^{\Omega} \sum_{j=1}^{\Omega} \ln(1 + f v_i) \ln(1 + f v_j) \sum_{\{W_k\}} f(\{W_k\}) \, (W_i - t\phi_i)(W_j - t\phi_j) \\
&= t \sum_{i=1}^{\Omega} \sum_{j=1}^{\Omega} (\phi_i \delta_{ij} - \phi_i \phi_j) \ln(1 + f v_i) \ln(1 + f v_j) \\
&= t \left[\sum_{i=1}^{\Omega} \phi_i \ln(1 + f v_i)^2 - \left(\sum_{i=1}^{\Omega} \phi_i \ln(1 + f v_i) \right)^2 \right]
\end{aligned} \tag{S19}$$

where δ_{ij} is the Kronecker delta. This result makes use of the known variance and covariance of the multinomial distribution—namely, $\mathrm{Var}(W_i) = t\phi_i(1 - \phi_i)$ and $\mathrm{Cov}(W_i, W_j) = t\phi_i\phi_j$ for $i \neq j$. These results for μ_t and σ_t^2 are identical to those derived in Appendix A.
